# Four New Species of *Sistotrema* (Hydnaceae, Cantharellales) from China

**DOI:** 10.3390/jof12060433

**Published:** 2026-06-13

**Authors:** Yu-Jin Cui, Jian-Zhao Qi, Guang-Yu Zeng, Rui Xing, Ying-Da Wu, Yu-Cheng Dai, Heng Zhao, Yuan Yuan

**Affiliations:** 1 School of Ecology and Nature Conservation, Beijing Forestry University, Beijing 100083, China; cuiyujin1126@bjfu.edu.cn (Y.-J.C.); zgy257@163.com (G.-Y.Z.); yuchengdai@bjfu.edu.cn (Y.-C.D.); zhaoheng181@mails.ucas.ac.cn (H.Z.); 2 Center of Edible Fungi, Northwest A&F University, Xianyang 712100, China; qjz@nwafu.edu.cn; 3 Guangxi Forestry Science Research Institute, Nanning 530002, China; 4 Northwest Institute of Plateau Biology, Chinese Academy of Sciences, Xining 810008, China; xingrui@nwipb.cas.cn; 5 Key Laboratory of Ministry of Emergency Management for Forest and Grassland Fire Risk Prevention, China Fire and Rescue Institute, Beijing 102202, China; wydbjfu@163.com; 6 CAS Key Laboratory of Forest Ecology and Silviculture, Institute of Applied Ecology, Chinese Academy of Sciences, Shenyang 110016, China

**Keywords:** new species, taxonomy, wood-decaying fungi, corticioid, poroid

## Abstract

Wood-inhabiting fungi play important ecological roles in forest ecosystems by participating in wood decomposition and nutrient cycling and represent a highly diverse group within Basidiomycota. In this study, four new wood-inhabiting species of *Sistotrema* from China are described and illustrated based on morphological examination and phylogenetic analyses of ITS, nLSU, nuc-SSU, and RPB2 sequence data. *Sistotrema armandii* is characterized by a hypochnoid hymenial surface, ventricose to suburniform basidia, and long sterigmata. *Sistotrema caeruleogriseum* is separated by its slightly tuberculate hymenial surface, subclavate to suburniform basidia with 4-sterigmata, and subglobose to globose basidiospores. *Sistotrema luteum* is typified by angular to irregular pores, thin and slightly fimbriate dissepiments, and globose basidiospores. *Sistotrema tenuissimum* is distinguished by a farinaceous hymenial surface, a monomitic hyphal system with thin- to slightly thick-walled generative hyphae, and barrel-shaped to suburniform basidia with 4 or 6 long sterigmata. The four new species and their phylogenetically related and morphologically similar species are discussed. This study expands the current knowledge of species diversity and phylogenetic relationships within the genus *Sistotrema* in China.

## 1. Introduction

The genus *Sistotrema* Fr., typified by *Sistotrema confluens* Pers., was established by Fries in 1821 and belongs to the family Hydnaceae within Cantharellales (Agaricomycetes, Basidiomycota). Species of *Sistotrema* are characterized by a monomitic hyphal system with clamped generative hyphae, often containing oily inclusions, urniform basidia bearing 4–8 sterigmata, and smooth, thin-walled basidiospores. The basidiomata are highly variable, ranging from resupinate to pileate or stipitate forms, with hymenophores that may be smooth, grandinioid, hydnoid, or poroid [1]. However, although the type species exhibits a terrestrial habit with stipitate basidiomata, most species of *Sistotrema* produce resupinate basidiomata on decayed wood [1,2,3].

As a widely distributed genus, species of *Sistotrema* have traditionally been regarded as wood-decaying fungi and are common components of wood-inhabiting fungal communities in forest ecosystems [4]. For example, *S. brinkmannii* (Bres.) J. Erikss. has been recorded on the bark of *Cedrus deodara*, whereas *S. porulosum* Hallenb. has been found on the bark of *Pinus wallichiana* and on dead and decaying logs of *C. deodara* [5]. However, recent molecular phylogenetic and ecological studies have demonstrated that the ecological diversity of the genus is broader than previously recognized. In addition to saprotrophic species associated with decayed wood, several *Sistotrema* species have been confirmed to form ectomycorrhizal associations with plants [6,7]. These findings suggest that species of *Sistotrema* occupy diverse ecological niches and exhibit considerable ecological differentiation.

Molecular phylogenetic studies have supported the placement of *Sistotrema* within Hydnaceae (Cantharellales, Basidiomycota) [8,9]. However, the genus remains polyphyletic in its current circumscription [6,9,10]. To address this issue, Münzenberger et al. [11] proposed the concept of the “core *Sistotrema*–*Hydnum* group”, which includes the type species of both genera [12]. Consequently, Sugawara et al. [7] restricted *Sistotrema* sensu stricto to the pileate-stipitate species *S. confluens* and *S. subconfluens* L.W. Zhou, while the remaining resupinate taxa were retained within *Sistotrema* sensu lato.

In recent years, numerous new species of *Sistotrema* have been reported worldwide, particularly from East Asia [3,7,13,14,15,16]. However, the number of accepted species within the genus has varied considerably among studies. Early studies estimated that *Sistotrema* comprised approximately 60 species [1,2,7], whereas more recent studies have recognized 111 species [3] or even more than 125 taxa. Currently, more than 220 names have been registered under *Sistotrema* in the MycoBank database (https://www.mycobank.org/, accessed on 30 March 2026). Several wood-inhabiting taxa have recently been described from China and Japan, indicating considerable hidden diversity within the genus and suggesting that East Asia represents an important center of diversity for *Sistotrema*. In addition, *Sistotrema bulbilliferum* G.G. Barreto & Gusmão was recently described from the bark of *Eucalyptus grandis* in Brazil, representing the first report of *Sistotrema* associated with *Eucalyptus* [17]. These findings suggest that the species diversity and host range of *Sistotrema* remain insufficiently explored.

Nevertheless, the taxonomy and species diversity of wood-inhabiting species of *Sistotrema* remain insufficiently understood, particularly in East Asia. The considerable morphological variability within the genus further complicates species delimitation. During investigations of wood-inhabiting fungi in several provinces of China, several specimens of *Sistotrema* were collected. Based on morphological characteristics and phylogenetic analyses of ITS, nLSU, nuc-SSU, and RPB2 sequence data, these specimens are described here as four new species.

## 2. Materials and Methods

### 2.1. Morphological Studies

The specimens examined in this study were dried basidiomata deposited in the Fungarium of the Institute of Microbiology, Beijing Forestry University (BJFC). No living cultures were obtained. The morphological descriptions were based on field notes and voucher specimens, following the methods outlined in previous studies [18,19,20]. Micro-morphological data and illustrations were obtained from the dried specimens and observed under a light microscope, following Miettinen et al. [21] and Wu et al. [18]. Sections were studied at magnification up to 1000× using a Nikon Eclipse 80i microscope and phase contrast illumination (Nikon, Tokyo, Japan). Descriptions of microscopic features and measurements were made from slide preparations stained with KOH, Cotton Blue and Melzer’s reagent (Macklin Biochemical Co., Ltd., Shanghai, China). The following abbreviations are used: KOH = 5% potassium hydroxide; CB = Cotton Blue; CB+ = cyanophilous in Cotton Blue; CB− = acyanophilous in Cotton Blue; IKI = Melzer’s reagent; IKI– = neither amyloid nor dextrinoid in Melzer’s reagent; L = mean basidiospore length (arithmetic average of basidiospores); W = mean basidiospore width (arithmetic average of basidiospores); Q = variation in the L/W ratios between specimens studied; *n* (a/b) = number of basidiospores (a) measured from the given number of specimens (b). Spores were measured from sections cut from the tubes or hymenial surface. In presenting the variation in basidiospore size, 5% of the measurements from each end of the range were excluded, and the excluded values are given in parentheses. Colour terms follow Petersen [22].

### 2.2. DNA Extraction, Amplification and Sequencing

A CTAB rapid plant genome extraction kit (DN14, Aidlab Biotechnologies Co., Ltd., Beijing, China) was used to extract total DNA from dried specimens, followed by the polymerase chain reaction (PCR) according to the manufacturer’s instructions with some modifications [23,24]. The internal transcribed spacer (ITS) and nuclear large subunit rDNA (nLSU) were amplified with primer pairs ITS5/ITS4 and LR0R/LR7 [25,26]. The nuclear small subunit rDNA (nuc-SSU) region was amplified with primer pairs NS1 and NS4 [25]. Part of RNA polymerase II second largest subunit (RPB2) region was amplified with primer pairs fRPB2-5F and fRPB2-7R [27].

The PCR procedure for ITS was as follows: initial denaturation at 95 °C for 3 min, followed by 35 cycles at 94 °C for 40 s, 54 °C for 45 s and 72 °C for 1 min, and a final extension of 72 °C for 10 min. The PCR procedure for nLSU and nuc-SSU was as follows: initial denaturation at 94 °C for 1 min, followed by 34 cycles of denaturation at 94 °C for 30 s, annealing at 58 °C for nLSU and 52 °C for nuc-SSU for 1 min and extension at 72 °C for 1.5 min, and a final extension at 72 °C for 10 min. The PCR procedure for RPB2 was initial denaturation at 94 °C for 2 min, followed by 10 cycles at 94 °C for 40 s, 60 °C for 40 s and 72 °C for 1.5 min, then followed by 37 cycles at 94 °C for 45 s, 55 °C for 1.5 min and 72 °C for 1.5 min, and a final extension of 72 °C for 10 min. The final PCR volume was 30 μL; each tube contained 1 μL of each primer, 1 μL extracted DNA, 12 μL ddH_2_O and 15 μL 2 × EasyTaq PCR Supermix (TransGen Biotech Co., Ltd., Beijing, China). The PCR products were purified and sequenced at the Beijing Genomics Institute (BGI), China, with the same primers as used in PCR. Newly generated sequences were deposited in GenBank. All sequences analyzed in this study are listed in Table 1.

### 2.3. Sequence Alignment

In this study, newly generated sequences were checked for ambiguous bases and assembled in BioEdit v.7.7.1.0 [45]. The newly generated sequences, together with additional sequences retrieved from GenBank, were aligned using MAFFT v.7 with the E-INS-i algorithm [46]. The resulting alignments were manually inspected and adjusted in BioEdit. The aligned datasets were concatenated and converted into different formats in Mesquite v.3.2 [47]. Ambiguously aligned regions were excluded, and gaps were manually adjusted to optimize the alignments prior to phylogenetic analyses.

### 2.4. Phylogenetic Analyses

The combined ITS, nLSU, nuc-SSU, and RPB2 dataset was used to infer the phylogenetic placement of the newly collected specimens. Refer to the criteria to select *Platygloea disciformis* (Voucher: AFTOL-ID 710) as outgroup [7]. The best-fit evolutionary model for the combined dataset was selected using ModelFinder v.1.6.12 [48] under the Akaike Information Criterion (AIC), and GTR + I + G was selected for Bayesian analyses. Phylogenetic analyses followed the methods of Barreto et al. [17] and Zhou et al. [13]. Maximum Likelihood (ML) analyses were performed using RAxML-HPC BlackBox v.8.2.12 on the CIPRES Science Gateway [49]. Branch support for ML analyses was estimated with 1000 bootstrap replicates. Bayesian Inference (BI) analyses and Bayesian posterior probabilities (BPPs) were conducted using MrBayes 3.1.2 [50]. Four Markov chains were run for 2,400,000 generations until the average standard deviation of split frequencies reached below 0.01, and trees were sampled every 1000 generations. The first 25% of sampled trees were discarded as burn-in, and the remaining trees were used to construct a majority-rule consensus tree and calculate Bayesian posterior probabilities. All phylogenetic trees were visualized using FigTree v.1.4.3 (http://tree.bio.ed.ac.uk/software/figtree/, accessed on 1 April 2026). Branches with ML bootstrap values ≥ 75% and BPPs ≥ 0.95 were considered significantly supported, while ML bootstrap values ≥ 50% and BPPs ≥ 0.90 are shown in the phylogenetic tree from ML analysis, respectively.

Newly generated sequences were compared with sequences in the NCBI GenBank database using Blast searches to identify closely related taxa.

## 3. Results

### 3.1. Molecular Phylogeny Studies

The combined ITS, nLSU, nuc-SSU, and RPB2 dataset comprised 107 sequences representing 58 taxa, including newly generated sequences and additional sequences retrieved from GenBank based on previous studies. The concatenated alignment contained 4360 characters, including gaps, of which positions 1–854 corresponded to ITS, 855–2245 to nLSU, 2246–3373 to nuc-SSU, and 3374–4360 to RPB2.

**Figure 1:**
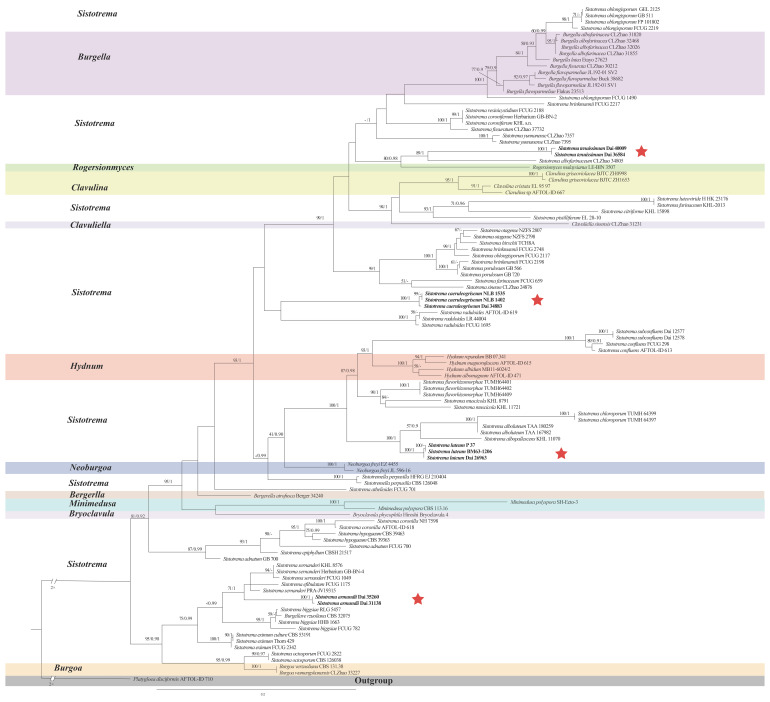
Phylogeny of *Sistotrema* by ML analysis based on the combined ITS, nLSU, nuc-SSU, and RPB2 dataset. Branches are labeled with Maximum Likelihood bootstrap values ≥ 50% and Bayesian Posterior Probabilities ≥ 0.90. Newly described species are in bold and marked with red stars. Species belonging to the same genus are indicated by the same color. The scale bar represents the estimated number of substitutions per site.

**Figure A1:**
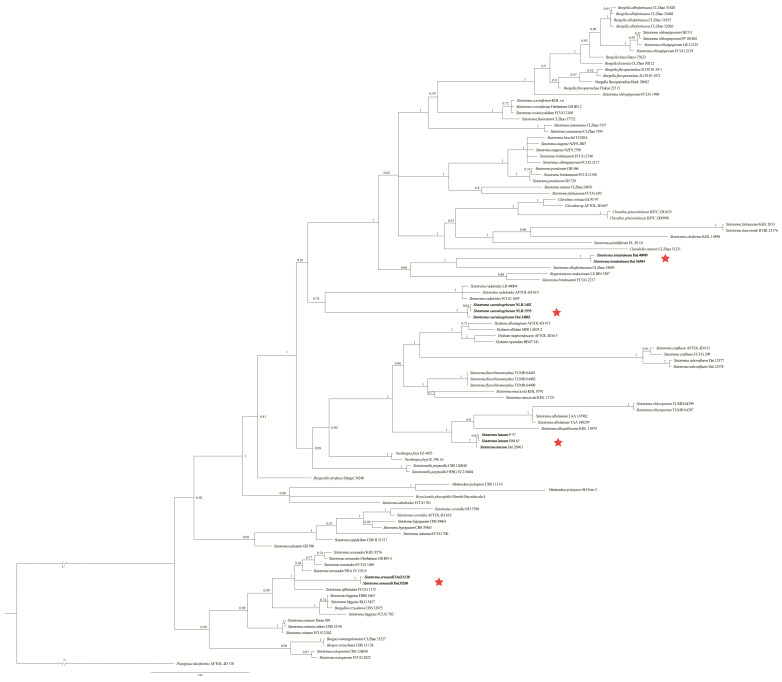
Phylogeny of *Sistotrema* by BI analysis based on the combined ITS, nLSU, nuc-SSU, and RPB2 dataset. Branches are labeled with Bayesian Posterior Probabilities. Newly described species are in bold and marked with red stars. The scale bar represents the estimated number of substitutions per site.

**Figure A2:**
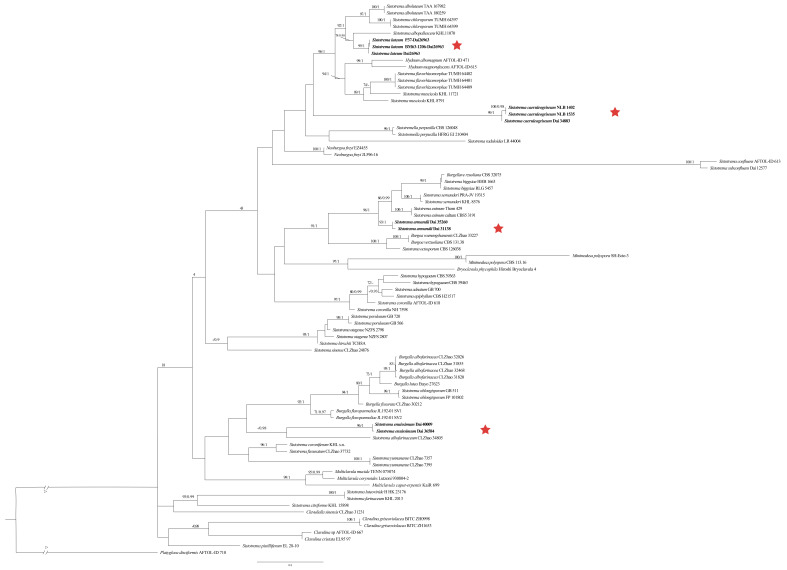
Phylogeny of *Sistotrema* by ML analysis based on the ITS dataset. Branches are labeled with Maximum Likelihood bootstrap values ≥ 50% and Bayesian Posterior Probabilities ≥ 0.90. Newly described species are in bold and marked with red stars. The scale bar represents the estimated number of substitutions per site.

**Figure A3:**
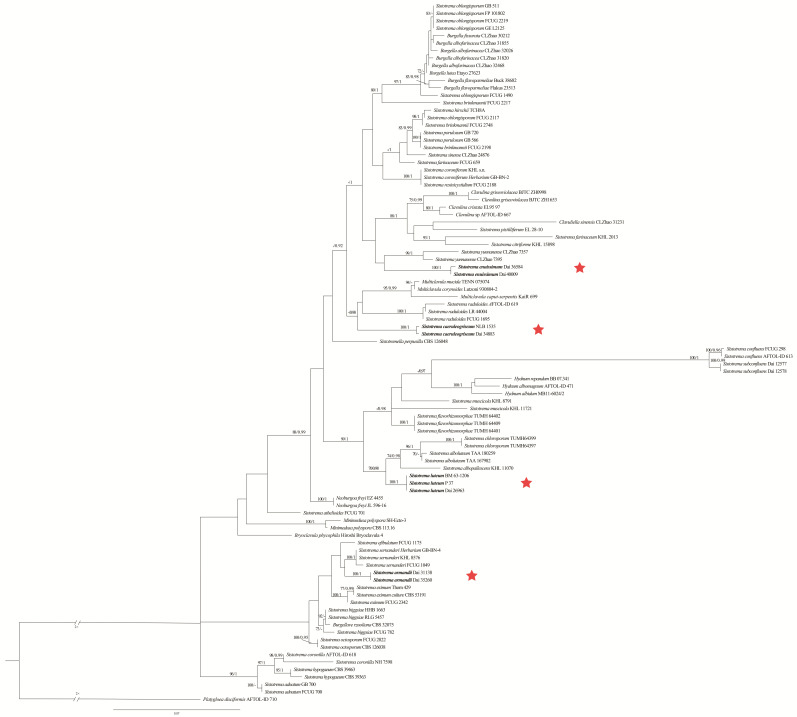
Phylogeny of *Sistotrema* by ML analysis based on the nLSU. Branches are labeled with Maximum Likelihood bootstrap values ≥ 50% and Bayesian Posterior Probabilities ≥ 0.90. Newly described species are in bold and marked with red stars. The scale bar represents the estimated number of substitutions per site.

Maximum Likelihood (ML) and Bayesian Inference (BI) analyses generated similar topologies with only minor differences in statistical support values. Therefore, only the ML tree is presented (Figure 1), with ML bootstrap values (≥50%) and Bayesian posterior probabilities (≥0.90) indicated at the nodes. The BI topology is provided in Appendix A (Figure A1). The Bayesian analysis reached convergence with an average standard deviation of split frequencies of 0.008972. The phylogenetic placements of the newly described species were also consistently revealed in the single-locus ITS and nLSU phylogenies (Figure A2 and Figure A3).

The phylogenetic analyses confirmed that the newly collected specimens represent four distinct lineages within *Sistotrema* (Figure 1). These lineages are described herein as *Sistotrema armandii*, *S. tenuissimum*, *S. luteum*, and *S. caeruleogriseum*.

In addition, the top three BLAST (https://blast.ncbi.nlm.nih.gov/Blast.cgi) matches for the newly generated ITS sequences, excluding uncultured sequences, are summarized in Table A1 to facilitate sequence comparison.

### 3.2. Taxonomy

The main morphological characteristics of the new species and related species in *Sistotrema* are provided in Table 2.

**Figure 2:**
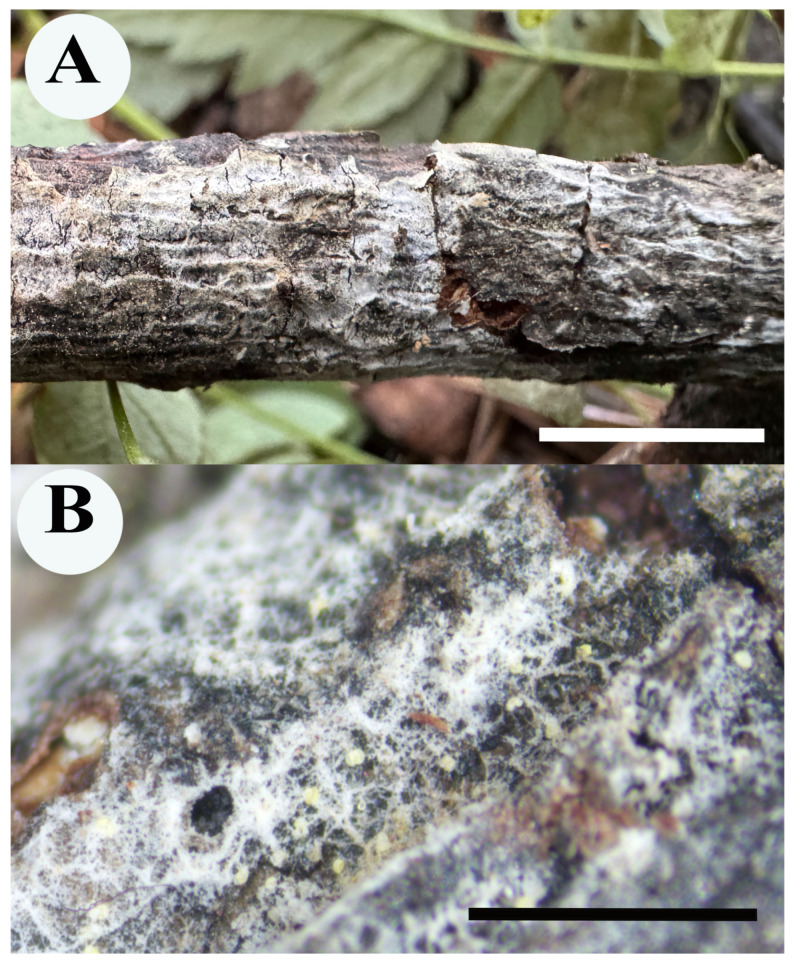
Basidiomata of ***Sistotrema armandii*** (Dai 35260, holotype). Scale bars: 1 cm (**A**); 1 mm (**B**).

**Figure 3:**
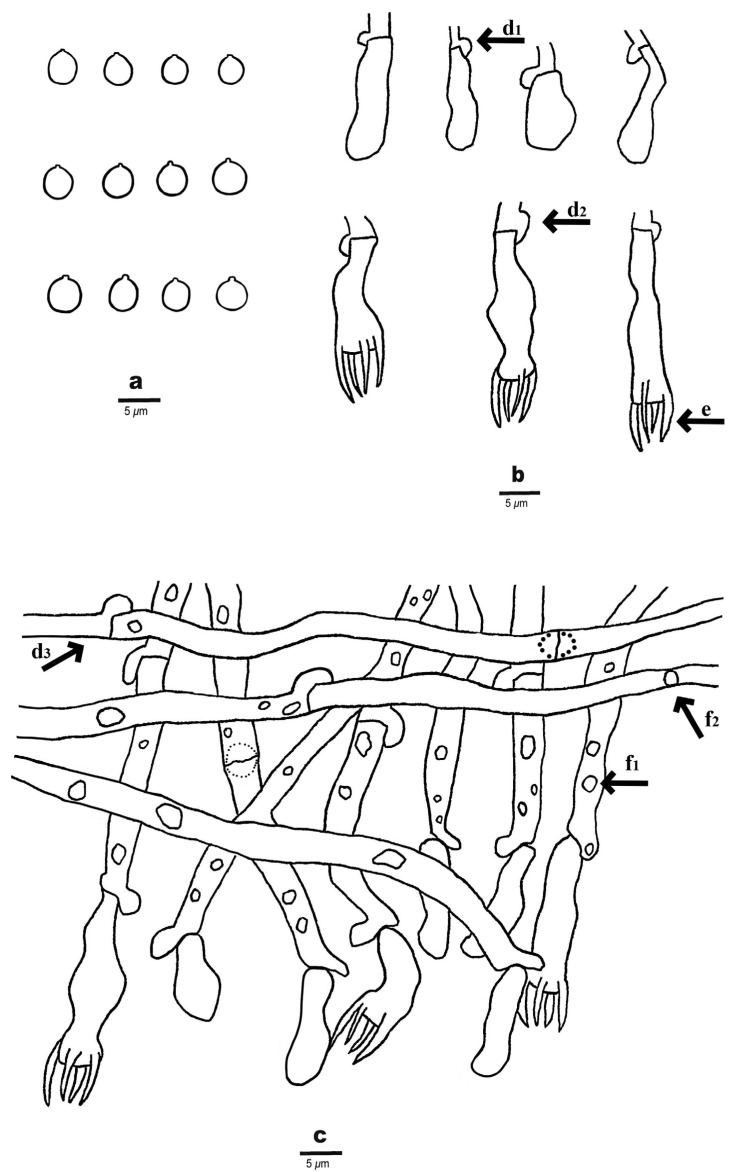
Microscopic structures of ***Sistotrema armandii*** (Dai 35260, holotype). (**a**) Basidiospores. (**b**) Basidia and basidioles. (**c**) A section of basidiomata. (**d_1_**–**d_3_**) Clamp connection. (**e**) Sterigmata. (**f_1_**,**f_2_**) Oily content.

***Sistotrema armandii* Y.J. Cui, Rui Xing, H. Zhao & Y.C. Dai** (Figure 2 and Figure 3)

**MycoBank no.** 863850

**Etymology**: *Armandii*, refers to the species growing on *Pinus armandii*.

**Holotype**: China, Qinghai, Xunhua County, Mengdatianchi Nature Reserve, longitude 102.6828 E, latitude 35.8011 N, elevation 2300 m, on fallen branch of *Pinus armandii*, 19 August 2025, Dai 35260 (holotype, BJFC 056521).

**Description**:

Basidiomata annual, resupinate, adnate, strongly attached to the wood, coriaceous, without odor or taste when fresh, becoming hard coriaceous upon drying, up to 5.5 cm long, 1 cm wide, 150 µm thick. Hymenial surface smooth, hypochnoid, white when fresh, turning white to cream (4A2) upon drying. Sterile margin narrow, thinning out, white, indeterminate.

Hyphal system monomitic, generative hyphae with clamp connections, usually with oily content, colorless, thin-walled, unbranched, slightly flexuous, interwoven, 2–4 μm in diam; IKI−, CB+; tissues unchanged in KOH.

Cystidia and cystidioles absent. Basidia clavate to suburniform, thin-walled, with 4 sterigmata and a basal clamp connection, 15–20 × 3.5–5.5 μm, sterigmata large, up to 5 μm long; basidioles abundant, clavate to pyriform, slightly smaller than basidia.

Basidiospores subglobose to globose, colorless, thin-walled, smooth, IKI−, CB−, 2.5–3.5(–4) × 2–3.2(–3.5) μm L = 3.14 µm, W = 2.92 µm, Q = 1.05–1.10 μm (*n* = 60/2).

**Distribution and ecology**: *Sistotrema armandii* is known from Northwest China (Qinghai), and grows on fallen branches of *Pinus armandii*, with its fruiting bodies observed in August and October.

**Additional specimen examined**: China, Qinghai, Xunhua County, Mengdatianchi Nature Reserve, longitude 102.6860 E, latitude 35.8119 N, elevation 2000 m, on fallen branch of *Pinus armandii*, 11 October 2024, Dai 31138 (BJFC 051397).

**Notes**: In the phylogenetic analyses, *Sistotrema armandii* is closely related to *S. sernanderi* (Litsch.) Donk and *S. efibulatum* (J. Erikss.) Hjortstam (Figure 1), and all species have monomitic hyphae system and resupinate basidiomata with smooth surface [1]. However, *S. sernanderi* is readily distinguished from *S. armandii* by its huge cystidia (50–80 × 5–8 µm vs. absent) [1] and longer basidiospores (5–7 × 2–3 µm vs. 2.5–3.5 × 2–3.2 µm) [1]. *S. efibulatum* differs from *S. armandii* by its longer basidiospores (5–6 × 2.5–3 µm vs. 2.5–3.5 × 2–3.2 µm) [1].

*S. sinense* is morphologically similar to *S. armandii* by a white and smooth surface, with almost the same wide hyphae. (2–4 µm in diam) [13]. However, the basidia of *S. sinense* is shorter than those of *S. armandii* (8–13.5 × 3–5.5 µm vs. 15–20 × 3.5–5.5) [13], and unrelated to *S. armandii* in phylogeny (Figure 1).

**Figure 4:**
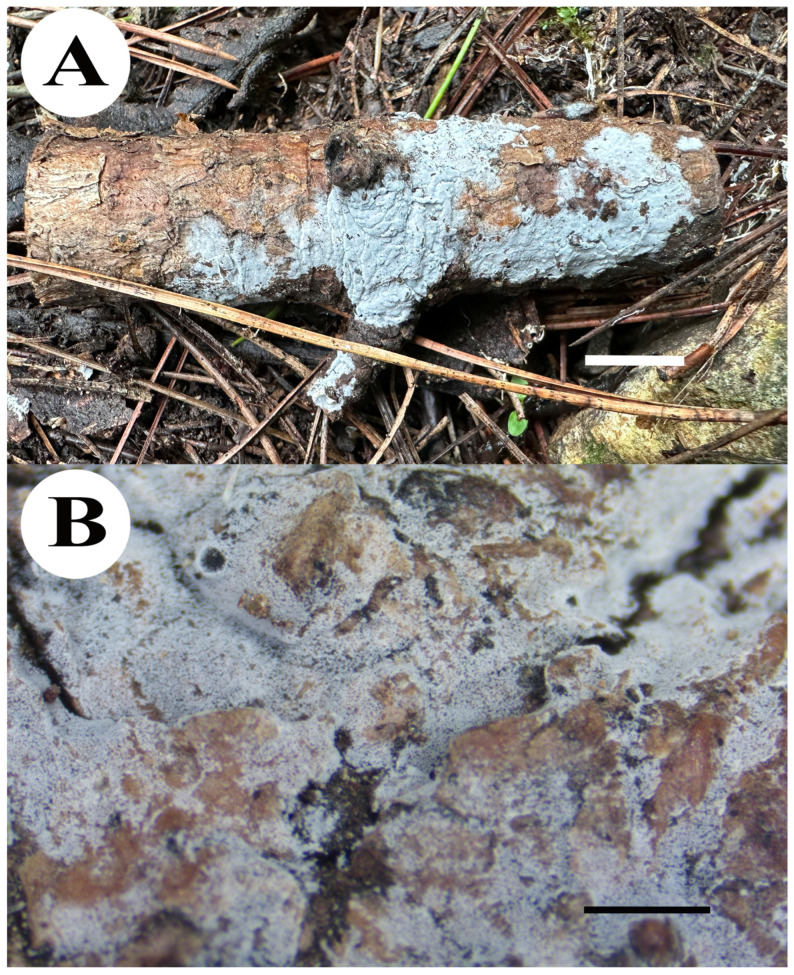
Basidiomata of ***Sistotrema caeruleogriseum*** (Dai 34883, holotype). Scale bars: 1 cm (**A**); 1 mm (**B**).

**Figure 5:**
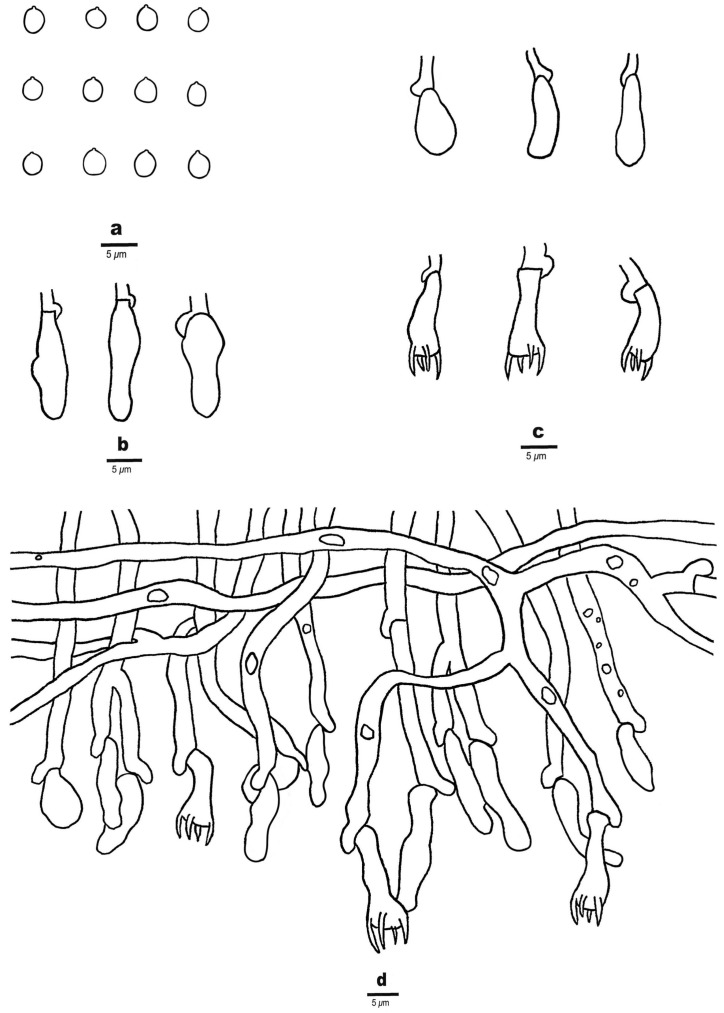
Microscopic structures of ***Sistotrema caeruleogriseum*** (Dai 34883, holotype). (**a**) Basidiospores. (**b**) Cystidioles. (**c**) Basidia and basidioles. (**d**) A section of basidiomata.

***Sistotrema caeruleogriseum* Y.J. Cui, J.Z. Qi, H. Zhao & Y.C. Dai** (Figure 4 and Figure 5)

**MycoBank no.** 863853

**Etymology**: *Caeruleogriseum*, refers to the species having a bluish gray hymenophore upon drying.

**Holotype**: China, Shaanxi, Ankang, Ningshan County, Huodingtang Experimental Forest Farm, longitude 108.4612 E, latitude 33.4354 N, elevation 1670 m, on a fallen branch of *Pinus tabuliformis*, 6 August 2025, Dai 34883 (holotype, BJFC 056144).

**Description**:

Basidiomata annual, resupinate, adnate, leathery, without odor or taste when fresh, becoming ceraceous to membranous upon drying, up to 5.5 cm long, 2 cm wide, 120 µm thick. Hymenial surface slightly tuberculate, white when fresh, turning to bluish grey (20C4) upon drying. Sterile margin narrows to almost absent.

Hyphal system monomitic, generative hyphae with clamp connections, with oily content, colorless, thin-walled, branched, slightly flexuous, interwoven, 3–4.5 μm in diam; IKI−, CB+; tissues unchanged in KOH.

Cystidia absent; cystidioles present, fusiform, thin-walled, smooth, 10–16 × 3–5 µm. Basidia subclavate to suburniform, thin-walled, with 4 sterigmata and a basal clamp connection, 10–15 × 2.6–5 μm; basidioles abundant, barrel-shaped to subclavate, slightly smaller than basidia.

**Distribution and ecology**: *Sistotrema caeruleogriseum* is known from Northwest China (Shaanxi) and Western Australia (Perth). In China, it grows on fallen branches of *Pinus tabuliformis*, and its fruiting bodies are observed in August.

Basidiospores subglobose to globose, colorless, thin-walled, smooth, IKI−, CB−, (1.9–)2–3 × (1.9–)2–2.7(–3) μm L = 2.35 µm, W = 2.23 µm, Q = 1.05 (*n* = 30/1).

**Notes**: *Sistotrema caeruleogriseum* and *S. raduloides* (P. Karst.) Donk. are closely related in the phylogenies. Both species share a monomitic, thin-walled hyphal system and similarly wide hyphae (3–4.5 µm vs. 2–6 µm) [1]. However, *S. raduloides* can be easily distinguished from *S. caeruleogriseum* by its longer basidiospores (7–9 × 3–3.5 µm vs. 2–3 × 2–2.7 µm) [1] and does not have cystidioles (absent vs. 10–16 × 3–5 µm) [1].

*S. albofarinaceum* Qiang Li & C.L. Zhao morphologically similar to *S. caeruleogriseum* in having a white hymenial surface, a monomitic, thin-walled hyphal system, and similar basidial dimensions (12–18.5 × 2.5–5 µm vs. 10–15 × 2.6–5 µm) [1]. In contrast, *S. albofarinaceum* differ markedly in basidiospore shape (ellipsoid vs. subglobose to globose) [1] and are not closely related phylogenetically (Figure 1).

**Figure 6:**
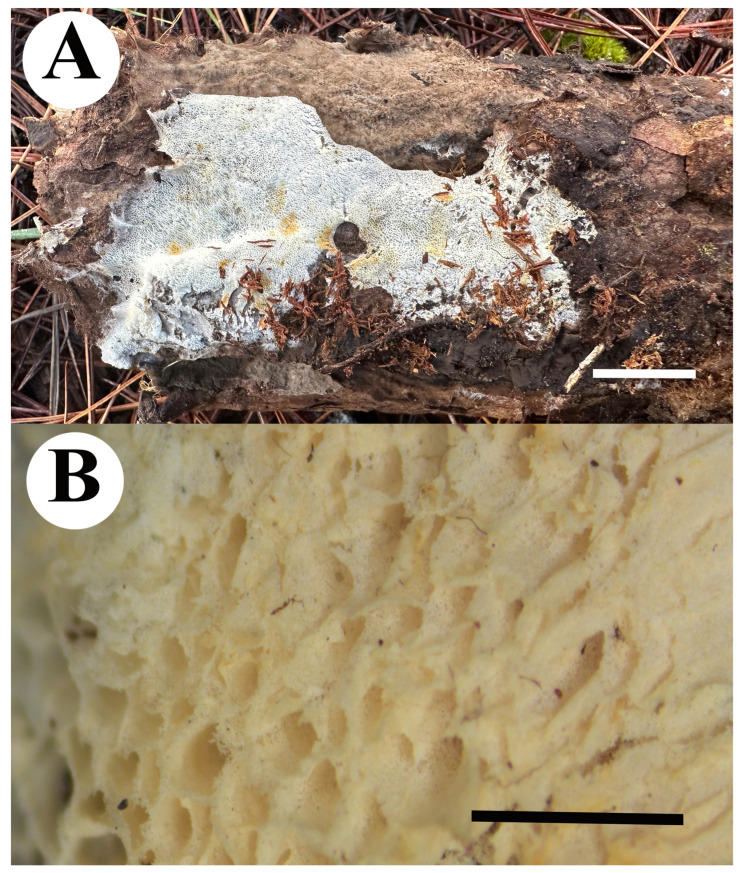
A Basidioma of ***Sistotrema luteum*** (Dai 26963, holotype). Scale bars: 1 cm (**A**); 1 mm (**B**).

**Figure 7:**
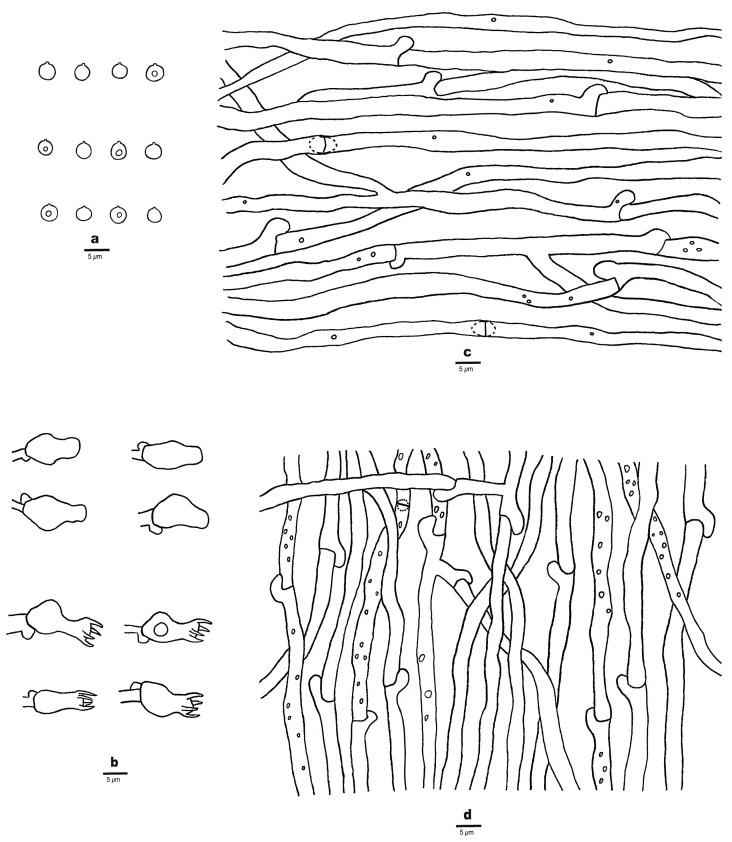
Microscopic structures of ***Sistotrema luteum*** (Dai 26963, holotype). (**a**) Basidiospores. (**b**) Basidia and basidioles. (**c**) Hyphae from context. (**d**) Hyphae from trama.

***Sistotrema luteum* Y.J. Cui, J.Z. Yuan Yuan, Y.D. Wu & Y.C. Dai** (Figure 6 and Figure 7)

**MycoBank no.** 863852

**Etymology**: *Luteum*, refers to the hymenophore of the species, which becomes orange-yellow when bruised.

**Holotype**: China, Xizang, Linzhi, Chayu County, Forests along road to Xiachayu town, longitude 97.3439 E, latitude 28.6231 N, elevation 2200 m, on charred wood of *Pinus yunnanensis*, 27 October 2023, Dai 26963 (holotype, BJFC 044514).

**Description**:

Basidiomata annual, resupinate, adnate, soft, without odor or taste when fresh, becoming soft leathery upon drying, up to 6 cm long, 3.5 cm wide, and 1.5 mm thick, margin thinning out, white, minutely fimbriate, up to 0.6 cm wide. Pore surface white to cream (4A2), becoming orange-yellow (4A8) when bruised, cream when dry, pores angular to irregular, 3–4 per mm, dissepiments thin, slightly fimbriate. Tubes concolorous with pore surface, up to 1 mm long. Subiculum white, paler contrast with tubes, homogeneous, fairly dense texture, up to 0.5 mm thick.

Hyphal system monomitic, generative hyphae with clamp connections, with oily content, colorless, thin-walled, branched, slightly flexuous, interwoven, 2–4 μm in diam; IKI−, CB+; tissues unchanged in KOH.

Contextual hyphae colorless, thin-walled, occasionally branched, sometimes with small oil drops, with frequent clamp connections, more or less straight, regularly arranged, IKI−, CB+.

Tramal hyphae hyaline, thin-walled, frequently branched, frequently with small oil drops, with abundant clamp connections, more or less straight, subparallel along the tubes, 2–4 μm in diam.

Cystidia and cystidioles absent. Basidia suburniform to urniform, rarely with a medium oil drop, thin-walled, with 4 sterigmata and a basal clamp connection, 11–15 × 3–5 μm; basidioles of similar shape to basidia, but slightly smaller. Basidiospores globose, colorless, thin-walled, smooth, sometimes with one oil drop, IKI−, CB−, (1.8–)2–2.5 µm in diam, Q = 1 (*n* = 30/1).

**Distribution and ecology**: *Sistotrema luteum* is known from Southwest China (Xizang) and Germany. In China, it grows on charred wood of *Pinus yunnanensis*, and its fruiting bodies are observed in October.

**Notes**: *Sistotrema luteum* is phylogenetically related to *S. alboluteum* (Bourdot & Galzin) Bondartsev & Singer, *S. albopallescens* Bourdot & Galzin and *S. chloroporum* (Figure 1). However, *S. alboluteum* is readily distinguished from *S. luteum* by its larger basidiospores (4.5–6 µm in diam vs. 2–2.5 µm in diam) [1]. *S. albopallescens* differs from *S. luteum* by its angular pores and subglobose spores. And *S. albopallescens* occurrence in Canada further suggests that it is a distinct species [51].

In addition, *S. chloroporum* is also morphologically similar to *S. luteum*, sharing a poroid, white to cream hymenial surface, angular pores, and a monomitic, thin-walled hyphal system with similarly wide hyphae (2.5–4 µm vs. 2–4 µm) [7]. However, it differs in basidiospore shape (Subglobose to broadly ellipsoid vs. Globose) [7] and has bigger basidia (15.5–27 × 7–9 µm vs. 11–15 × 3–5 µm) [7].

**Figure 8:**
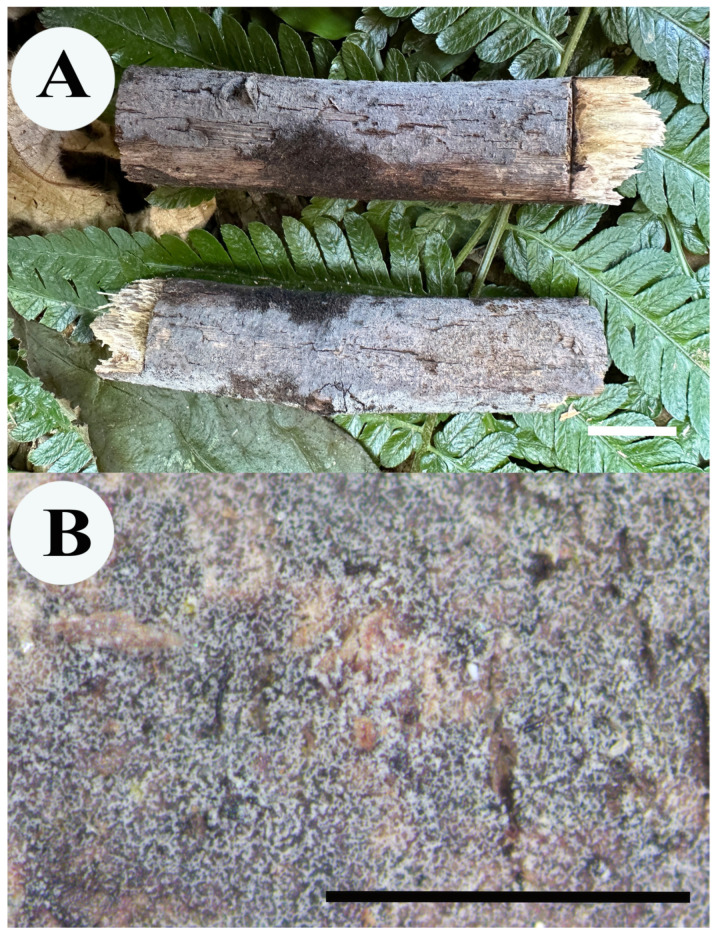
Basidiomata of ***Sistotrema tenuissimum*** (Dai 40009, holotype). Scale bars: 1 cm (**A**); 1 mm (**B**).

**Figure 9:**
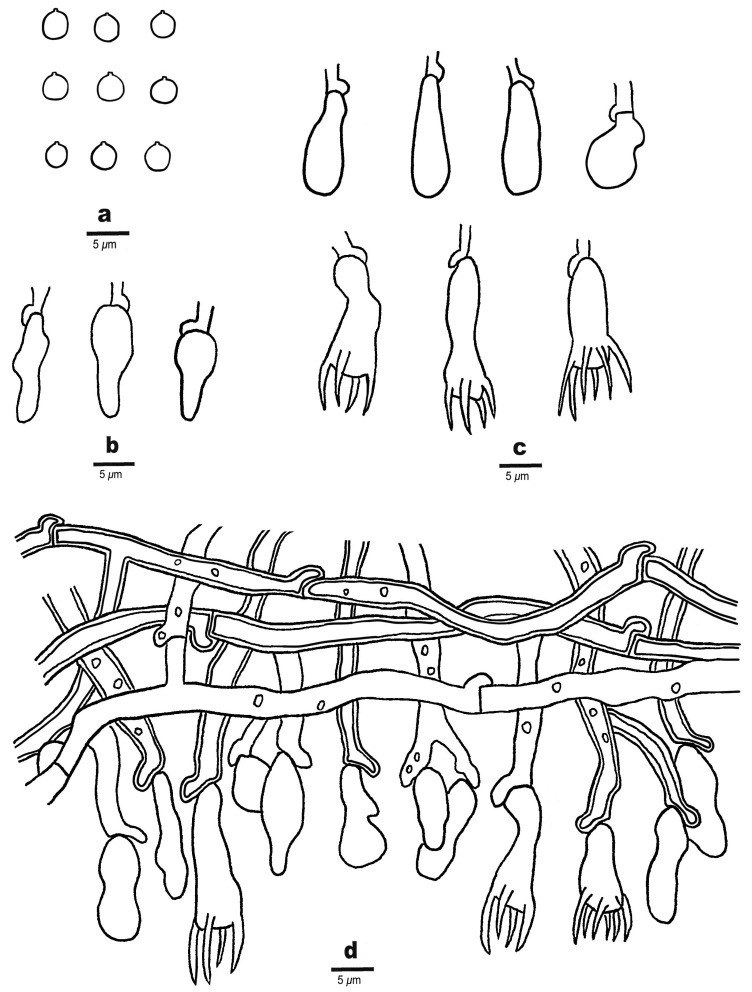
Microscopic structures of ***Sistotrema tenuissimum*** (Dai 40009, holotype). (**a**) Basidiospores. (**b**) Cystidioles. (**c**) Basidia and basidioles. (**d**) A section of basidiomata.

***Sistotrema tenuissimum* Y.J. Cui, G.Y. Zeng, H. Zhao & Y.C. Dai** (Figure 8 and Figure 9)

**MycoBank no.** 863851

**Etymology**: *Tenuissimum*, refers to the species having very thin basidiomata.

**Holotype**: China, Guangxi Auto. Reg., Rongshui County, Sirong Forest, longitude 109.1981 E, latitude 25.1411 N, elevation 200 m, on fallen angiosperm branch, 27 October 2025, Dai 40009 (holotype, BJFC 061266).

**Description**:

Basidiomata annual, resupinate, adnate, soft pellicular, without odor or taste when fresh, rigid upon drying, up to 14 cm long, 2.5 cm wide, 90 µm thick. Hymenial surface smooth, farinaceous, white when fresh, turning to bluish gray (20C4) upon drying. Sterile margin narrow, thinning out, white, indeterminate.

Hyphal system monomitic, generative hyphae with clamp connections, usually with oily content, colorless, thin- to thick-walled with a wide lumen, occasionally branched, flexuous, interwoven, 1.5–3 μm in diam; IKI−, CB−; tissues darkening in KOH.

Cystidia absent; cystidioles present, fusiform to lageniform, thin-walled, smooth, 10–14 × 3–5 µm. Basidia barrel-shaped to suburniform, thin-walled, with 4 or 6 sterigmata and a basal clamp connection, 10–15 × 3.5–5.5 μm, sterigmata large, sterigmata up to 5 μm long; basidioles abundant, clavate to subpyriform, slightly smaller than basidia.

Basidiospores subglobose to globose, colorless, thin-walled, smooth, IKI−, CB+, (2–)2.2–3.1(–3.3) × (2–)2.2–3(–3.3) μm, L = 2.68 µm, W = 2.60 µm, Q = 1.03–1.07 (*n* = 60/2).

**Additional specimen examined**: China, Guangxi, Rongshui County, Jiuwanshan Nature Reserve, longitude 108.6733 E, latitude 25.2056 N, elevation 1200 m, on dead bamboo culm, 14 May 2025, Dai 36584 (BJFC 057843).

**Distribution and ecology**: *Sistotrema tenuissimum* is known from South China (Xizang) and grows on fallen angiosperm branches and dead bamboo culm, with its fruiting bodies observed in May and October.

**Notes**: *Sistotrema tenuissimum* is phylogenetically related to *S. albofarinaceum* (Figure 1). Both species share a monomitic hyphal system and resupinate basidiomata with smooth, white surface. However, the latter does not have cystidioles (absent vs. 10–14 × 3–5 µm) [14] and they differ morphologically in basidiospore shape. (Ellipsoid vs. subglobose to globose) [14].

*S. yunnanense* L.Q. Cai & C.L. Zhao is morphologically similar to *S. tenuissimum*, both of which were collected in China and share a resupinate, smooth, farinaceous, white hymenial surface as well as a monomitic hyphal system. Nevertheless, *S. yunnanense* produces larger basidiospores (4.5–6.5 × 3–4 µm vs. 2.2–3.1 × 2.2–3 µm) [3]. Furthermore, these two species form independent clades in the phylogeny (Figure 1).

## 4. Discussion

Molecular phylogenetic analyses based on ITS, nLSU, nuc-SSU, and RPB2 sequence data confirmed that the newly collected specimens represent four distinct lineages within *Sistotrema*. Combined with morphological evidence, these lineages are herein described as *S. armandii*, *S. caeruleogriseum*, *S. luteum*, and *S. tenuissimum*. The discovery of these new taxa further demonstrates the substantial hidden diversity of wood-inhabiting species within *Sistotrema*. Despite recent advances in the taxonomy of the genus, the generic circumscription and phylogenetic relationships of many resupinate species remain insufficiently resolved [52,53,54,55], highlighting the need for additional specimen sampling and multi-loci phylogenetic data. In particular, East Asia, especially southwestern China, appears to represent an important center of diversity for *Sistotrema* species.

Notably, the strongly supported sister relationship between *Sistotrema farinaceum* (KHL 2013) and *S. luteoviride* (H HK23176) is accompanied by identical ITS sequences. This pattern suggests that these taxa may represent a species complex. However, confirmation of this hypothesis will require re-examination of the voucher specimens together with additional molecular data. This case further demonstrates that the phylogenetic relationships and species boundaries within *Sistotrema* remain insufficiently resolved and require comprehensive taxonomic revision. Resolving the taxonomic affinities within the genus will require extensive sampling, additional sequence data, and detailed morphological re-examination of reference specimens.

Species of *Sistotrema* are widely distributed from temperate to tropical regions, although most available records are concentrated in the Northern Temperate Zone [56]. Analyses of GBIF [56] occurrence data (6893 records) revealed that collections from the Northern Temperate Zone (6245 records) greatly outnumbered those from tropical regions (86 records) and the Southern Temperate Zone (269 records), suggesting either a genuine biogeographic pattern or potential sampling bias. In the Northern Temperate Zone, most collections were made between August and November, with a pronounced peak in September, which may be associated with seasonal climatic conditions favorable for basidiomata production. In contrast, tropical collections showed a more even distribution throughout the year, possibly reflecting relatively stable environmental conditions. These patterns indicate that the diversity and distribution of *Sistotrema* species are likely influenced by both ecological factors and uneven collecting efforts, particularly in underexplored tropical and subtropical regions.

Some species of *Sistotrema* have been confirmed as ectomycorrhizal fungi. Characterization of ectomycorrhizae is important for understanding the ecological status and taxonomic relationships of fungi [57,58,59]. Although numerous ectomycorrhizal descriptions have been published [57,60], only a few species within *Sistotrema* sensu lato have been investigated using molecular identification of field-collected ectomycorrhizae or in vitro synthesis [6,11,61]. Sugawara et al. [57] provided detailed descriptions of the basidiomata and ectomycorrhizae of *S. chloroporum* R. Sugaw., N. Maek., Sotome & N. Endo and *S. flavorhizomorphae* R. Sugaw., N. Shiras., N. Maek. & N. Endo, revealing considerable differences in mycorrhizal morphology and host preference between the two species. *Sistotrema chloroporum* was associated mainly with broadleaf hosts in Betulaceae and Fagaceae, whereas *S. flavorhizomorphae* showed a preference for *Pinus* species. Interestingly, both *S. armandii* and *S. caeruleogriseum* were collected in association with *Pinus* species, which may indicate a similar host preference in these taxa. These findings suggest that ecological differentiation and host preference may contribute to speciation within the genus.

In addition to their ecological importance, species of *Sistotrema* may also possess potential agricultural applications. Ye et al. [62] reported that a *Sistotrema* species dominant in the rhizosphere of blueberry (*Vaccinium* spp.) was positively associated with vigorous plant growth. The fungus exhibited iron-solubilizing and nitrogen-fixing capabilities and inhibited soil nitrification, thereby increasing soil nitrogen and available iron contents. Inoculation experiments further demonstrated that the fungus promoted blueberry growth and enhanced tolerance to nutrient deficiency and drought stress. These findings suggest that the ecological functions of *Sistotrema* species may extend beyond wood decay and ectomycorrhizal symbiosis, with promising potential for the development of biofertilizers and other agricultural applications.

Although some of the proposed species in this study, such as *S. caeruleogriseum* and *S. luteum*, are currently represented by only a single collection, we do not consider this to undermine their taxonomic validity. Additional sequences retrieved from GenBank, originating from independent collections and different geographic regions, clustered consistently with our specimens in the phylogenetic analyses and formed well-supported monophyletic lineages. These results provide independent molecular evidence supporting the recognition of these taxa as distinct species. Nevertheless, we acknowledge that the currently available sampling remains limited for certain species, and therefore caution should be exercised when interpreting their full morphological variability, ecological preferences, and geographic distributions. Additional collections, particularly from broader geographic areas and diverse ecological habitats, will be necessary to further refine species boundaries and improve our understanding of species diversity within *Sistotrema*.

In recent years, substantial progress has been made in the study of wood-inhabiting fungal diversity worldwide, particularly in China [63,64,65,66]. Nevertheless, the diversity of many corticioid and wood-inhabiting fungi remains insufficiently explored [67,68]. In this study, four new species of *Sistotrema* were identified based on morphological characteristics and multi-locus phylogenetic analyses. The discovery of these taxa expands the currently known diversity of *Sistotrema* and further highlights the largely unexplored fungal diversity of China. Continued field investigations and integrative taxonomic studies will undoubtedly reveal additional undescribed diversity within the genus and related fungal groups. In conclusion, the four new species described in this study expand the known diversity of *Sistotrema* and further highlight the largely unexplored fungal diversity of China.

## Figures and Tables

**Table 1:** A list of taxa information and GenBank accession numbers of sequences used in this study.

Species Name	Country	Voucher	GenBank Accession No.				References
			*ITS*	*nLSU*	*nuc-SSU*	*RPB2*	
*Bergerella atrofusca* (type)	Austria	BR Berger 34240	—	MN902070	—	—	[28]
*Bryoclavula phycophila* (type)	Japan	Hiroshi Bryoclavula4	OQ791465	OQ791464	—	—	Genbank
*Burgella albofarinacea*	China	CLZhao 31820	PQ758751	PQ758759	—	—	[13]
*B. albofarinacea*	China	CLZhao 32468	PQ758754	PQ758762	—	—	[13]
*B. albofarinacea*	China	CLZhao 32026	PQ758753	PQ758761	—	—	[13]
*B. albofarinacea*	China	CLZhao 31855	PQ758752	PQ758760	—	—	[13]
*B. fissurata*	China	CLZhao 30212	PQ758749	PQ758757	—	—	[13]
*B. flavoparmeliae* (type)	USA	Flakus 23513	—	KC336074	—	—	[29]
*B. flavoparmeliae*	Bolivia	Buck 38682	—	DQ915469	DQ915455	—	[29]
*B. flavoparmeliae*	USA	JL192-01 SV1	OR471304	—	OR470928	—	[30]
*B. flavoparmeliae*	USA	JL192-01 SV2	OR471305	—	OR470929	—	[30]
*B. lutea*	Bolivia	Etayo 27623	KC336076	KC336075	—	—	[29]
*B. wumengshanensis*	China	CLZhao 33227	PQ758755	—	—	—	[13]
*B. verzuoliana* (type)	Italy	CBS 131.38	AB972781	—	—	—	[31]
*B. verzuoliana*	Czech Republic	CBS 32075	OR471176	OR470988	OR470812	—	[30]
*Clavuliella sinensis* (type)	China	CLZhao 31231	PQ758750	PQ758758	—	—	[13]
*Clavulina cristata* (type)	Sweden	EL 95_97	AY463398	AY586648	—	—	[32]
*Clavulina* sp.	USA	AFTOL-ID 667	DQ202266	AY745694	AY757265	DQ366286	[33], Genbank
*C. griseoviolacea*	China	BJTC ZH1653	PP835338	PP835352	—	PP889524	[34]
*C. griseoviolacea*	China	BJTC ZH0998	PP835334	PP835347	—	PP889520	[34]
*Hydnum albidum*	Thailand	MB11-6024/2	—	AY293186	AY293136	—	[35]
*H. albomagnum*	USA	AFTOL-ID 471	DQ218305	AY700199	AY665777	DQ234553	[36], Genbank
*H. magnorufescens*	USA	AFTOL-ID 615	DQ218306	—	DQ457636	DQ366288	[33]
*H. repandum*	France	BB 07.341	—	KF294643	—	KF294720	[37]
*Minimedusa. polyspora* (type)	USA	CBS 113.16	MH854646	MH866167	—	—	[38]
*M. polyspora*	China	SH-Ecto-3	MG833806	MG833798	—	—	Genbank
*Neoburgoa freyi* (type)	Vietnam	JL596-16	KX423755	KX423755	—	OR474000	[30,39]
*N. freyi*	Canada	EZ 4455	OR471314	OR471068	OR470936	—	[30]
*Platygloea disciformis*	USA	AFTOL-ID 710	DQ234556	AY629314	DQ234563	DQ234554	[7]
*Rogersiomyces malaysianus*	Poland	LE-BIN 3507	KT779285	—	—	—	[40]
*Sistotremella perpusilla*	USA	CBS 126048	MH864061	MH875516	—	—	[38]
*S. perpusilla*	Panama	HFRG EJ 210404	OL828790	—	—	—	Genbank
*Sistotrema confluens* (type)	Canada	FCUG 298	—	DQ898711	DQ898726	DQ898761	[8]
*S. confluens*	Canada	AFTOL-ID 613	DQ267125	AY647214	AY757260	DQ381837	[33,36]
*S. adnatum*	Sweden	FCUG 700	—	DQ898699	DQ898725	DQ898763	[8]
*S. adnatum*	Sweden	GB 700	OR464426	OR460895	—	OR473859	[30]
*S. albofarinaceum*	China	CLZhao 34805	PV569135		—	—	[14]
*S. alboluteum*	Canada	TAAM 167982	AY463467	AY586713	—	—	[32]
*S. alboluteum*	Sweden	TAAM 180259	AJ606042	AJ606042	—	—	[6]
*S. albopallescens*	Canada	KHL 11070	AM259209	AM259210	—	—	[6]
***S. armandii***	China	Dai 31138	PZ395126	PZ395121	PZ417093	PZ428812	Present study
***S. armandii***	China	Dai 35260	PZ395127	PZ395122	PZ417094	PZ428813	Present study
*S. athelioides*	Japan	FCUG 701	—	DQ898700	—	—	[8]
*S. biggsiae*	Sweden	FCUG 782	—	DQ898697	—	—	[8]
*S. biggsiae*	USA	RLG5457	OR464437	OR460907	OR464298	OR473732	[30]
*S. biggsiae*	USA	HHB 1663	OR464396	OR460881	OR464261	OR473731	[30]
*S. brinkmannii*	Canada	FCUG 2217		DQ898709	DQ898715	DQ898755	[8]
*S. brinkmannii*	Canada	FCUG 2198		DQ898705	DQ898713	DQ898753	[8]
*S. brinkmannii*	Canada	FCUG 2748		DQ898704	DQ898714	DQ898752	[8]
***S. caeruleogriseum***	China	Dai 34883	PZ395128	PZ395123	PZ417095	—	Present study
***S. caeruleogriseum***	Australia	NLB 1402	MT537018		—	—	Genbank
***S. caeruleogriseum***	Australia	NLB 1535	MT537104	MT537104	—	—	Genbank
*S. chloroporum*	Sweden	TUMH 64399	NR178117	LC642057	LC642034	—	[7]
*S. chloroporum*	Japan	TUMH 64397	LC642031	LC642055	—	—	[7]
*S. citriforme*	Sweden	KHL15898	KF218962	KF218962	—	—	[41]
*S. coroniferum*	Canada	GB-BN-2	—	AM259215	—	—	[6]
*S. coroniferum*	Netherlands	KHL s.n.	KF218968	KF218968	—	—	[41]
*S. coronilla*	USA	NH 7598	AF506475	AF506475	—	—	[32]
*S. coronilla*	USA	AFTOL-ID 618	DQ397337	DQ457641	AY757259	DQ381838	[33], Genbank
*S. efibulatum*	Canada	FCUG 1175	—	DQ898696	DQ898721	—	[8]
*S. epiphyllum*	Canada	CBS H-21517	NR155795	—	—	—	Genbank
*S. eximum*	Finland	Thorn429	OR464436	AF393076	—	AY218518	[30,42]
*S. eximum*	Japan	CBS 531.91	MH862275	MH873956	—	—	[38]
*S. eximum*	Canada	FCUG 2342	—	DQ898695	AY757261	DQ898762	[8], Genbank
*S. farinaceum* (Type)	Japan	FCUG 659	—	DQ898707	DQ898718	DQ898756	[8]
*S. farinaceum*	Australia	KHL 2013	KF218963	KF218963	—	—	[41]
*S. fissuratum* (Type)	China	CLZhao 37732	PV569136	—	—	—	[14]
*S. flavorhizomorphae* (Type)	Japan	TUMH 64409	NR178118	NG153858		LC667371	[7]
*S. flavorhizomorphae*	Finland	TUMH 64401	LC642038	LC642059	—	—	[7]
*S. flavorhizomorphae*	Sweden	TUMH 64402	LC642040	LC642060	—	—	[7]
*S. hirschii*	USA	TCH8A	OR464366	OR460864	OR464241	OR473748	[30]
*S. hypogaeum*	Finland	CBS 394.63	MH858314	MH869926	—	—	[38]
*S. hypogaeum*	Australia	CBS 393.63	MH858313	MH869925	—	—	[38]
*S. luteoviride*	Sweden	H HK23176	NR158892	—	—	—	[41]
***S. luteum***	China	Dai 26963	PZ395129	PZ428930	—	—	Present study
***S. luteum***	Germany	P 37	FR838002	FR838002	—	—	Genbank
***S. luteum***	Germany	BM 63-1206	AM747290	AM747290	—	—	Genbank
*S. muscicola*	Canada	KHL 8791	AF506474	AF506474	—	—	[32]
*S. muscicola*	USA	KHL 11721	AJ606040	AJ606040	—	—	[6]
*S. oblongisporum*	Sweden	FCUG 1490	—	DQ898702	DQ898716	DQ898758	[8]
*S. oblongisporum*	Sweden	GB 511	OR471173	OR470985	OR470809	OR474007	[30]
*S. oblongisporum*	USA	FP101802	OR464432	OR460902	OR464292	—	[30]
*S. oblongisporum*	Canada	GEL2125		DQ898728	DQ898738	DQ898767	[8]
*S. oblongisporum*	Canada	FCUG 2219		DQ898701	DQ898719	DQ898757	[8]
*S. oblongisporum*	Canada	FCUG 2117		DQ898703	DQ898717	DQ898759	[8]
*S. octosporum*	USA	FCUG 2822	—	DQ898698	DQ898722	DQ898764	[8]
*S. octosporum*	Finland	CBS 126038	MH864053	MH875510	—	—	[38]
*S. otagense*	New Zealand	NZFS 2807	MN007030	—	—	—	Genbank
*S. otagense*	New Zealand	NZFS 2798	MN007029	—	—	—	Genbank
*S. pistilliferum*	Canada	EL 28-10	KF218964	KF218964	—	—	[41]
*S. porulosum*	Canada	GB 566	OR464431	OR460901	OR464291	—	[30]
*S. porulosum*	Canada	GB 720	OR464435	OR460905	OR464296	—	[30]
*S. raduloides*	Sweden	AFTOL-ID 619	—	AY647213	AY757262	—	[36], Genbank
*S. raduloides*	USA	LR 44004	KF218969	KF218969	—	—	[41]
*S. raduloides*	Canada	FCUG 1695		DQ898710	DQ898727	DQ898765	[8]
*S. resinicystidium*	China	FCUG 2188	—	DQ898708	DQ898720	DQ898760	[8]
*S. sernanderi*	China	GB-BN-4	—	AM259219	—	—	[6]
*S. sernanderi*	China	KHL 8576	—	AF506476	—	—	[43]
*S. sernanderi*	Sweden	FCUG 1049	—	AY647215	AY757264	—	[6]
*S. sernanderi*	Czech Republic	PRA-JV19315	OM104976	—	—	—	[44]
*S. sinense*	China	CLZhao 24876	PQ758748	PQ758756	—	—	[13]
*S. subconfluens*	China	Dai 12577	JX076812	JX076810	—	—	[15]
*S. subconfluens*	Sweden	Dai 12578	—	JX076811	—	—	[15]
***S. tenuissimum***	China	Dai 36584	PZ395130	PZ395124	PZ417096	PZ428810	Present study
***S. tenuissimum***	China	Dai 40009	PZ395131	PZ395125	PZ417097	PZ428811	Present study
*S. yunnanense*	USA	CLZhao 7357	ON817194	ON810362	—	—	[3]
*S. yunnanense*	UK	CLZhao 7395	ON817195	ON810363	—	—	[3]

Notes: New species are in bold.

**Table 2:** Morphological comparison of four new *Sistotrema* species with their morphologically similar or phylogenetically related species.

Species Name	Hymenial Surface	Cystidia/Cystidioles (µm)	Basidia (µm)	Hyphae (µm)	Basidiospores (µm)	References
*Sistotrema. albofarinaceum*	Smooth/ white	Absent	Subcylindrical/ 4-sterigmata/ 12–18.5 × 2.5–5	Monomitic/ thin-walled/ 2–3/ with clamps	Ellipsoid/ 2.5–3.2 × 2–2.5	[14]
*S. alboluteum*	Poroid/ yellow or vitellinous/ pores angular/ 1–4 per mm	Absent	Urniform/ 2 or 4-sterigmata/ 20–30 × 7–10	Monomitic/ thin-walled/ 2–8/ with clamps	Globose/ 4.5–6 in diam	[1]
*S. albopallescens*	Poroid/ white, creamy, or finally fulvous/ pores angular	_	Basidia with distal portion strongly expanded at the summit	_	Spores subglobose/ 2.5–4.5 × 2–4	[51]
***S. armandii***	Smooth, hypochnoid/ white	Absent	Clavate to suburniform/ 4-sterigmata/ 15–20 × 3.5–5.5	Monomitic/ thin-walled/ 2–4/ with clamps	Subglobose to globose/ 2.5–3.5 × 2–3.2	Present study
***S. caeruleogriseum***	Slightly tuberculate/ white	Cystidioles fusiform/ thin-walled/ smooth/ 10–16 × 3–5	Subclavate to suburniform/ 4-sterigmata/ 10–15 × 2.6–5	Monomitic/ thin-walled/ 3–4.5/ with clamps	Subglobose to globose/ 2–3 × 2–2.7	Present study
*S. chloroporum*	Poroid/ whitish to cream/ pores angular to round/ 2–4 per mm	Absent	Urniform/ 4 or 6-sterigmata/ 15.5–27 × 7–9	Monomitic/ thin-walled/ 2.5–4/ with clamps	Subglobose to broadly ellipsoid/ 4.5–6.5 × 3.5–6	[7]
*S. efibulatum*	Smooth/ greyish-white	Absent	Urniform/ normally with 6 sterigmata; no basal clamp/ 18–22 × 5–7 µm	Monomitic/ more or less thickened walls/ 4–6/ without clamps	Ovoid to ellipsoid/ 5–6 × 2.5–3	[1]
***S. luteum***	Poroid/ white to cream/ pores angular to irregular/ 3–4 per mm	Absent	Suburniform to urniform/ 4-sterigmata/ 11–15 × 3–5/	Monomitic/ thin-walled/ 2–4/ with clamps	Globose/ 2–2.5 in diam	Present study
*S. oblongisporum*	Smooth/ greyish–whitish grey	Absent	First rounded–ovoid, when mature urniform/ 6–8 sterigmata/ 15–18 × 4–6	Monomitic/ thin-walled/ 2.5–3.5/ with clamps	Suballantoid/ 5–6 × 1.5–2	[1]
*S. raduloides*	Lightcoloured with small papilliform aculei when young, the mature fungus pale buff, odontioid–hydnoid, with normally cylindrical, rarely irregular teeth	Absent	Urniform/ (4) 6 or 8-sterigmata/ 18–23 × 5–7	Monomitic/ thin-walled/ 2–6/ with clamps	Subcylindrical–subfusiform/ 7–9 × 3–3.5	[1]
*S. sernanderi*	Smooth or somewhat tuberculate/ white to pale cream	Cystidia (gloeocystidia) flexuose/ 50–80 × 5–8/ thin-walled	Narrowly urniform to cylindrical/ 4-sterigmata/ 15–30 × 3.5–6 µm	Monomitic/ thin or somewhat thickened walls/ 5–7/ with clamps	Narrowly ellipsoid–suboblong–suballantoid/ 5–7 × 2–3	[1]
*S. sinense*	Smooth/ white	Absent	Suburniform to urniform/ 4-sterigmata/ 8–13.5 × 3–5.5	Monomitic/ slightly thick-walled/ 2–4/ with clamps	Suballantoid to allantoid/ 3–4.5 × 1.5–2.5	[13]
***S. tenuissimum***	Smooth, farinaceous/ white	Cystidioles fusiform to lageniform/ thin-walled/ smooth/ 10–14 × 3–5	Barrel–shaped to suburniform/ 4 or 6 sterigmata/ 10–15 × 3.5–5.5	Monomitic/ thin- to thick-walled/ 1.5–3/ with clamps	Subglobose to globose/ 2.2–3.1 × 2.2–3	Present study

Notes: New species are in bold.

## Data Availability

The original contributions presented in this study are included in the article. Further inquiries can be directed to the corresponding author.

## Appendix A

**Table A1:** BLAST results of ITS sequences of the newly described *Sistotrema* species based on searches in GenBank.

Species Name	Locus	Rank	Matched Species	Voucher	Max Score	Query Cov. (%)	Identity (%)	Align. Length	Accession No.
*Sistotrema armandii*	ITS	1	*Sistotrema sernanderi*	17F012	612	100	91.43	644	MK968526
		2	*Sistotrema sernanderi*	447503	612	100	91.43	620	MW795655
		3	*Sistotrema sernanderi*	PUL F24585	612	100	91.21	660	MW448617
*Sistotrema caeruleogriseum*	ITS	1	*Sistotrema* sp.	NLB 1535	1153	100	99.37	1635	MT537104
		2	*Sistotrema* sp.	NLB 1402	1149	100	99.22	802	MT537018
		3	*Sistotrema* sp.	g1p36c	983	88	98.39	583	PV135204
*Sistotrema luteum*	ITS	1	*Sistotrema* sp.	P37	1070	100	99.83	1207	FR838002
		2	*Sistotrema* sp.	BM63-1206	1066	100	99.83	1196	FR838001
		3	*Sistotrema* sp.	UC2022930	1009	94	99.82	602	KP814241
*Sistotrema tenuissimum*	ITS	1	*Sistotrema* sp.	MO415150	604	97	87.14	632	OK346345
		2	*Sistotrema* sp.	FLAS-F-61125	599	91	88.16	595	MH399865
		3	*Sistotrema* sp.	MO415150	604	97	87.14	632	OK346345

## References

[1] Eriksson J., Hjortstam K., Ryvarden L. (1984). The Corticiaceae of North Europe.

[2] Bernicchia A., Gorjón S.P. (2010). Fungi Europaei 12: Corticiaceae s.l..

[3] Cai L.Q., Zhao C.L. (2023). Molecular phylogeny and morphology reveal a new wood-rotting fungal species, *Sistotrema yunnanense* sp. nov. from the Yunnan-Guizhou Plateau. Mycoscience.

[4] Lindner D.L., Burdsall H.H., Stanosz G.R. (2006). Species diversity of polyporoid and corticioid fungi in northern hardwood forests with differing management histories. Mycologia.

[5] Kaur M., Singh A.P., Dhingra G.S., Kaur R. (2018). The genus *Tylopilus* Fr. (Boletaceae, Basidiomycota) from district Shimla (Himachal Pradesh). KAVAKA.

[6] Nilsson R.H., Larsson K.H., Larsson E., Kõljalg U. (2006). Fruiting body-guided molecular identification of root-tip mantle mycelia provides strong indications of ectomycorrhizal associations in two species of *Sistotrema* (Basidiomycota). Mycol. Res..

[7] Sugawara R., Shirasuka N., Yamamoto T., Nagamune K., Oguchi K., Maekawa N., Sotome K., Nakagiri A., Ushijima S., Endo N. (2022). Two new species of *Sistotrema* s.l. (Cantharellales) from Japan with descriptions of their ectomycorrhizae. Mycoscience.

[8] Moncalvo J.M., Nilsson R.H., Koster B., Dunham S.M., Bernauer T., Matheny P.B., Porter T.M., Margaritescu S., Weiß M., Garnica S. (2006). The cantharelloid clade: Dealing with incongruent gene trees and phylogenetic reconstruction methods. Mycologia.

[9] Hibbett D.S., Bauer R., Binder M., Giachini A.J., Hosaka K., Justo A., Larsson E., Larsson K.-H., Lawrey J.D., Miettinen O., McLaughlin D., Spatafora J. (2014). 14 Agaricomycetes. Systematics and Evolution.

[10] Larsson K.H. (2007). Re-thinking the classification of corticioid fungi. Mycol. Res..

[11] Münzenberger B., Schneider B., Nilsson R.H., Bubner B., Larsson K.H., Hüttl R.F. (2012). Morphology, anatomy, and molecular studies of the ectomycorrhiza formed axenically by the fungus *Sistotrema* sp. (Basidiomycota). Mycol. Prog..

[12] Cao T., Hu Y.P., Yu J.R., Wei T.Z., Yuan H.S. (2021). A phylogenetic overview of the Hydnaceae (Cantharellales, Basidiomycota) with new taxa from China. Stud. Mycol..

[13] Zhou Q., Qian C.B., Zhang C.Y., Su Q.D., Li Y.L., Zhang S.H., Mu N., Xu T.M., Zhou H.M., Zhao C.L. (2025). Morphology and multigene phylogeny reveals five new species of Hydnaceae (Cantharellales, Basidiomycota) from China. MycoKeys.

[14] Wijesinghe S.N., Deng Y.L., Yuan Q., Zhou H.M., Wang L., Dai Y.F., Xu Y., Jiang N., AlOtibi F., Al-Sadi A.M. (2025). Mycosphere Notes 572–624: Exploring the hidden diversity of fungi and fungi-like taxa in different terrestrial microhabitats. Mycosphere.

[15] Zhou L.W., Qin W.M. (2013). *Sistotrema subconfluens* sp. nov. (Cantharellales, Basidiomycota) from Changbaishan Nature Reserve, Northeastern China. Mycoscience.

[16] Cui Y.J., Wu Y.D., Jiang Y.H., Zhu A.H., Wu F., Liu H.G., Dai Y.C., Yuan Y. (2025). Diversity of macrofungi in southeast Xizang 1. The wood decay fungi. Mycology.

[17] Barreto G.G., Cantillo T., Costa-Rezende D.H., Gusmão L.F.P. (2023). A new bulbil-forming species of *Sistotrema* (Cantharellales, Hydnaceae) from Brazil. Phytotaxa.

[18] Wu F., Zhou L.W., Vlasák J., Dai Y.C. (2022). Global diversity and systematics of Hymenochaetaceae with poroid hymenophore. Fungal Divers..

[19] Wang C.G., Dai Y.C., Kout J., Gates G.M., Liu H.G., Yuan Y., Vlasák J. (2025). Multi–gene phylogeny and taxonomy of *Physisporinus* (Polyporales, Basidiomycota). Mycosphere.

[20] Zhang Q.Y., Huang J.H., Ren J.L., Zhu L.H., Huang L. (2026). Morphological and phylogenetic analyses reveal a new genus and two new species of Hymenochaetales (Basidiomycota) from southeast China. MycoKeys.

[21] Miettinen O., Vlasák J., Rivoire B., Spirin V. (2018). *Postia caesia* complex (Polyporales, Basidiomycota) in temperate Northern Hemisphere. Fungal Syst. Evol..

[22] Petersen J.H. (1996). The Danish Mycological Society´s Colour-Chart.

[23] Sun Y.F., Costa-Rezende D.H., Xing J.H., Zhou J.L., Zhang B., Gibertoni T.B., Gates G., Glen M., Dai Y.C., Cui B.K. (2019). Multi-gene phylogeny and taxonomy of the brown-rot fungi: *Postia* (Polyporales, Basidiomycota) and related genera. Persoonia.

[24] Zhao H., Nie Y., Zong T.K., Wang K., Lv M.L., Cui Y.J., Tohtirjap A., Chen J.J., Zhao C.L., Wu F. (2023). Species diversity, updated classification and divergence times of the phylum Mucoromycota. Fungal Divers..

[25] White T.J., Bruns T., Lee S., Taylor J., Innis M.A., Gelfand D.H., Sninsky J.J., White T.J. (1990). Amplification and direct sequencing of fungal ribosomal RNA genes for phylogenetics. PCR Protocols, a Guide to Methods and Applications.

[26] Hopple J.S., Vilgalys R. (1999). Phylogenetic relationships in the mushroom genus *Coprinus* and dark-spored allies based on sequence data from the nuclear gene coding for the large ribosomal subunit RNA: Divergent domains, outgroups, and monophyly. Mol. Phylogenet. Evol..

[27] Matheny P.B. (2005). Improving phylogenetic inference of mushrooms with RPB1 and RPB2 nucleotide sequences (*Inocybe*, Agaricales). Mol. Phylogenet. Evol..

[28] Lawrey J.D., Sikaroodi M., Gillevet P.M., Diederich P. (2020). A new species of bulbil-forming lichenicolous fungi represents an isolated clade in the Cantharellales. Bryologist.

[29] Diederich P., Lawrey J.D., Capdet M., Pereira S., Romero A., Etayo J., Flakus A., Sikaroodi M., Ertz D. (2014). New lichen-associated bulbil-forming species of Cantharellales (Basidiomycetes). Lichenologist.

[30] Swenie R.A., Looney B., Ke Y.H., Rojas J.A., Cubeta M., Langer G., Vilgalys R., Matheny P. (2024). PacBio high-throughput multi-locus sequencing reveals high genetic diversity in mushroom-forming fungi. Mol. Ecol. Resour..

[31] Kiyuna T., An K.D., Kigawa R., Sano C., Miura S., Sugiyama J. (2015). “Black particles”, the major colonizers on the ceiling stone of the stone chamber interior of the Kitora Tumulus, Japan, are the bulbilliferous basidiomycete fungus *Burgoa anomala*. Mycoscience.

[32] Larsson K.H., Larsson E., Kõljalg U. (2004). High phylogenetic diversity among corticioid Homobasidiomycetes. Mycol. Res..

[33] Matheny P.B., Wang Z., Binder M., Curtis J.M., Lim Y.W., Nilsson R.H., Hughes K.W., Hofstetter V., Ammirati J.F., Schoch C.L. (2007). Contributions of rpb2 and tef1 to the phylogeny of mushrooms and allies (Basidiomycota, Fungi). Mol. Phylogenet. Evol..

[34] Cao T., Hu Y.P., Yu J.R., Wei T.Z., Yuan H.S. (2021). Three new species of the genus *Clavulina* (Hydnaceae, Cantharellales) from North China based on morphological and phylogenetic analysis. Mycologia.

[35] Binder M., Hibbett D.S., Larsson K.H., Larsson E., Langer E., Langer G. (2005). The phylogenetic distribution of resupinate forms across the major clades of mushroom-forming fungi (Homobasidiomycetes). Syst. Biodivers..

[36] Masumoto H., Degawa Y. (2020). *Bryoclavula phycophila* gen. et sp. nov. belonging to a novel lichenized lineage in Cantharellales (Basidiomycota). Mycol. Prog..

[37] Buyck B., Kauff F., Eyssartier G., Couloux A., Hofstetter V. (2014). A multilocus phylogeny for worldwide *Cantharellus* (Cantharellales, Agaricomycetidae). Fungal Divers..

[38] Vu D., Groenewald M., De Vries M., Gehrmann T., Stielow B., Eberhardt U., Al-Hatmi A., Groenewald J.Z., Cardinali G., Houbraken J. (2019). Large-scale generation and analysis of filamentous fungal DNA barcodes boosts coverage for kingdom Fungi and reveals thresholds for fungal species and higher taxon delimitation. Stud. Mycol..

[39] Lawrey J.D., Zimmermann E., Sikaroodi M., Diederich P. (2016). Phylogenetic diversity of bulbil-forming lichenicolous fungi in Cantharellales including a new genus and species. Bryologist.

[40] Psurtseva N.V., Zmitrovich I.V., Malysheva V.F. (2016). Taxonomy and developmental morphology of *Rogersiomyces malaysianus* comb. nov. (Cantharellales, Agaricomycetes). Botany.

[41] Kotiranta H., Larsson K.H. (2013). *Sistotrema luteoviride* sp. nov. (Cantharellales, Basidiomycota) from Finland. Acta Mycol..

[42] Binder M., Hibbett D.S. (2002). Higher-level phylogenetic relationships of Homobasidiomycetes (mushroom-forming fungi) inferred from four rDNA regions. Mol. Phylogenet. Evol..

[43] Larsson E., Larsson K.-H. (2003). Phylogenetic relationships of russuloid basidiomycetes with emphasis on aphyllophoralean taxa. Mycologia.

[44] Vondrák J., Svoboda S., Zíbarová L., Štenclová L., Mareš J., Pouska V., Košnar J., Kubásek J. (2023). Alcobiosis, an algal-fungal association on the threshold of lichenisation. Sci. Rep..

[45] Hall T.A. (1999). BioEdit: A user-friendly biological sequence alignment editor and analysis program for Windows 95/98/NT. Nucleic Acids Symp. Ser..

[46] Katoh K., Standley D.M. (2013). MAFFT multiple sequence alignment software version 7: Improvements in performance and usability. Mol. Phylogenet. Evol..

[47] Maddison W.P., Maddison D.R. Mesquite: A Modular System for Evolutionary Analysis 2017, Version 3.2. http://mesquiteproject.org.

[48] Kalyaanamoorthy S., Minh B.Q., Wong T.K.F., von Haeseler A., Jermiin L.S. (2017). ModelFinder: Fast model selection for accurate phylogenetic estimates. Nat. Methods.

[49] Miller M.A., Holder M.T., Vos R., Midford P.E., Liebowitz T., Chan L., Hoover P., Warnow T. 2009, The CIPRES Portals. http://phylo.org/sub_sections/portal.

[50] Ronquist F., Huelsenbeck J.P. (2003). MRBAYES 3: Bayesian phylogenetic inference under mixed models. Bioinformatics.

[51] Rogers D.P. (1944). The genera *Trechispora* and *Galzinia*. Mycologia.

[52] Zhou L.W., Vlasák J., Qin W.M., Dai Y.C. (2016). Global diversity and phylogeny of the *Phellinus igniarius* complex (Hymenochaetales, Basidiomycota) with the description of five new species. Mycologia.

[53] Hyde K.D., Noorabadi M.T., Thiyagaraja V., He M.Q., Johnston P.R., Wijesinghe S.N., Armand A., Biketova A.Y., Chethana K.W.T., Erdoğdu M. (2024). The 2024 Outline of Fungi and fungus-like taxa. Mycosphere.

[54] Zhao H., Cui Y.J., Guan Q.X., Wang K., Zhuang L., Zeng G.Y., Wei Y.L., Wu F., Yuan H.S. (2026). Global species diversity and distribution patterns within the order Hymenochaetales (Agaricomycetes, Basidiomycota). Mycosphere.

[55] Thiyagaraja V., Hyde K.D., Piepenbring M., Davydov E.A., Dai D.Q., Abdollahzadeh J., Bundhun D., Chethana K.W.T., Crous P.W., Gajanayake A.J. (2025). Orders of Ascomycota. Mycosphere.

[56] GBIF Occurrence Download. https://doi.org/10.15468/dl.xbspp4.

[57] Agerer R. (2006). Fungal relationships and structural identity of their ectomycorrhizae. Mycol. Prog..

[58] Moyersoen B., Weiß M. (2014). New Neotropical Sebacinales species from a *Pakaraimaea dipterocarpacea* Forest in the Guayana Region, Southern Venezuela: Structural diversity and phylogeography. PLoS ONE.

[59] Mrak T., Kühdorf K., Grebenc T., Štraus I., Münzenberger B., Kraigher H. (2017). *Scleroderma areolatum* ectomycorrhiza on *Fagus sylvatica* L.. Mycorrhiza.

[60] Bahram M., Põlme S., Kõljalg U., Tedersoo L. (2011). A single European aspen (*Populus tremula*) tree individual may potentially harbour dozens of *Cenococcum geophilum* ITS genotypes and hundreds of species of ectomycorrhizal fungi. FEMS Microbiol. Ecol..

[61] Bubner B.C., Morgner W.S., Münzenberger B. (2014). Proof of ectomycorrhizal status of *Sistotrema confluens* Pers., the type species of the polyphyletic genus *Sistotrema*. Mycol. Prog..

[62] Ye Y., Zhan X., Wang K., Zhong J., Liao F., Chen W., Guo W. (2023). A Symbiotic Fungus *Sistotrema* Benefits Blueberry Rejuvenation and Abiotic Stress Tolerance. J. Fungi.

[63] Wang C.G., Zhao H., Liu H.G., Zeng G.Y., Yuan Y., Dai Y.C. (2023). A multi-gene phylogeny clarifies species diversity, taxonomy, and divergence times of *Ceriporia* and other related genera in Irpicaceae (Polyporales, Basidiomycota). Mycosphere.

[64] Zhao H., Yuan H.S., Cui Y.J., Wang K., Wu F., Dai Y.C., Yuan Y. (2026). Global polypore diversity and distribution patterns. Fungal Divers..

[65] Cao Y.H., Zhang Q.Y. (2026). Molecular phylogeny and morphology reveal a new species of wood-inhabiting fungal *Dacrymyces* (Dacrymycetaceae, Dacrymycetes) from Eastern China. Phytotaxa.

[66] Bao D.F., Tian X.G., Samarakoon M.C., Karunarathna S.C., Luo Z.L., Han J.J., Wen T.C., He Z.J., Liu Z.H., Lu Y.Z. (2025). Biodiversity of lignicolous freshwater fungi from the Nanpan River Basin in Guizhou and Guangxi Provinces, China, with descriptions of fifteen species. Mycosphere.

[67] Zhang Q.Y., Liu H.G., Papp V., Zhou M., Dai Y.C., Yuan Y. (2023). New insights into the classification and evolution of *Favolaschia* (Agaricales, Basidiomycota) and its potential distribution, with descriptions of eight new species. Mycosphere.

[68] Wijayawardene N.N., Hyde K.D., Mikhailov K.V., Goto B.T., Santiago A.L.C.M.A., Tokarev Y.S., Elshahed M.S., Madrid H., Pires-Zottarelli C.L.A., Pawłowska J. (2025). Families of non-Dikarya fungi. Mycosphere.

